# How Curiosity Enhances Hippocampus-Dependent Memory: The Prediction, Appraisal, Curiosity, and Exploration (PACE) Framework

**DOI:** 10.1016/j.tics.2019.10.003

**Published:** 2019-12

**Authors:** Matthias J. Gruber, Charan Ranganath

**Affiliations:** 1 Cardiff University Brain Research Imaging Centre (CUBRIC), School of Psychology, Cardiff University, Cardiff, UK; 2 Center for Neuroscience, University of California, Davis, Davis, CA, USA; 3 Psychology Department, University of California, Davis, Davis, CA, USA

**Keywords:** curiosity, memory, hippocampus, prediction, appraisal, exploration

## Abstract

Curiosity plays a fundamental role for learning and memory, but the neural mechanisms that stimulate curiosity and its effect on memory are poorly understood. Accumulating evidence suggests that curiosity states are related to modulations in activity in the dopaminergic circuit and that these modulations impact memory encoding and consolidation for both targets of curiosity and incidental information encountered during curiosity states. To account for this evidence, we propose the Prediction, Appraisal, Curiosity, and Exploration (PACE) framework, which attempts to explain curiosity and memory in terms of cognitive processes, neural circuits, behavior, and subjective experience. The PACE framework generates testable predictions that can stimulate future investigation of the mechanisms underlying curiosity-related memory enhancements.

## Highlights

A fledgling line of curiosity research shows how states of curiosity enhance learning and retention.
To account for the current evidence, we propose the Prediction, Appraisal, Curiosity, and Exploration (PACE) framework, which integrates the emergent research on curiosity and draws on a range of ideas from psychology, cognitive neuroscience, and systems neuroscience.
PACE proposes that curiosity is triggered by significant prediction errors that are appraised. This cycle enhances memory encoding through increased attention, exploration, and information seeking and enhances the consolidation of information acquired while in a curious state through dopaminergic neuromodulation of the hippocampus.
The PACE framework provides a common reference scheme for future research on curiosity-mediated memory and generates testable predictions to stimulate further research.

## Understanding Curiosity as a Cognitive State

Curiosity is assumed to have a fundamental impact on learning and memory and thus has been a major topic of interest to educators [1]. Although early research on curiosity primarily focused on curiosity described as a stable tendency to experience curiosity (i.e., trait curiosity) [2], there has been increased interest in curiosity as a cognitive state (i.e., state curiosity) 3, 4, 5, 6. Researchers generally describe state curiosity as a motivational state that stimulates exploration and information seeking to reduce uncertainty 7, 8, 9, 10. State curiosity resembles states that fall under the umbrella of ‘reward motivation’, in the sense that information that resolves uncertainty can be seen as having a value comparable with other rewards 11, 12, 13, 14. Although several recent reviews have highlighted the importance of state curiosity in learning 2, 8, 9, 11, 13, this nascent field currently lacks a theoretical framework that attempts to explain curiosity and memory in terms of cognitive processes and their underlying neural circuits.

**Figure 1:**
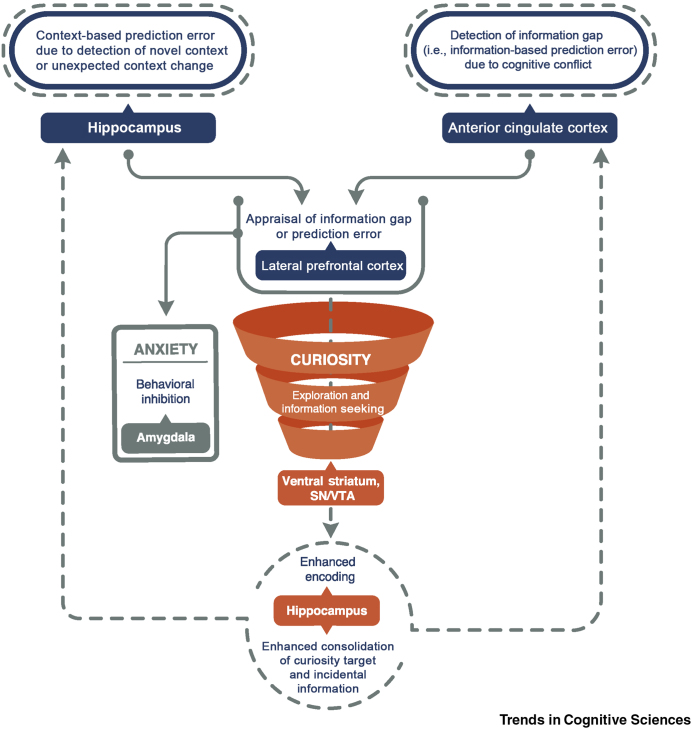
Key Figure. The Prediction, Appraisal, Curiosity, and Exploration (PACE) Framework We propose that the effects of curiosity and memory can be understood as emerging from a cycle that involves prediction errors, appraisal, curiosity, and exploration. The framework implies that there are different factors that can trigger curiosity and that curiosity also affects memory in multiple ways. In general, the PACE framework proposes that curiosity is elicited by context-based prediction errors supported by the hippocampus and information-based prediction errors (information gaps) supported by the anterior cingulate cortex (ACC). Within the PACE framework, prediction errors might not be sufficient to trigger curiosity or could even have the opposite effect and induce anxiety due to the uncertain state. We propose that prediction errors and information gaps trigger an appraisal process [supported by the lateral prefrontal cortex (PFC)] that determines one’s actions (i.e., inhibition or exploration) along with its subjective experience and underlying neural mechanisms (i.e., anxiety-related amygdalar processes or curiosity-related dopaminergic processes). If curiosity is sparked, curiosity enhances learning via increased attentional processes during information seeking and retention via enhanced memory consolidation. A PACE cycle will be completed once uncertainty is resolved and curiosity is satisfied by closure of an information gap. However, in many cases the presentation of the curiosity target information might elicit a further context- or information-based prediction error. Such further prediction errors will start a new PACE cycle, which can then further benefit memory and promote knowledge acquisition. The PACE framework stimulates future research on curiosity and memory along with testable predictions (Box 3).

In this Opinion article, we propose a framework that integrates emergent research on curiosity, drawing on a broad range of evidence and ideas from psychology 4, 15, 16, 17, cognitive neuroscience 3, 5, 6, 18, 19, and systems neuroscience 14, 20, 21. Specifically, we propose that the effects of curiosity and memory can be understood as emerging from a cycle that involves Prediction errors, Appraisal, Curiosity, and Exploration (PACE; Figure 1, Key Figure). This framework proposes that curiosity is first triggered by significant prediction errors that are appraised as an indicator of information that could be valuable in the future. This cycle enhances memory encoding through increased attention, exploration, and information seeking and enhances the consolidation of information acquired while in a curious state through dopaminergic neuromodulation of the hippocampus. The framework leads to testable predictions and provides a common reference scheme for future research on curiosity and its relationship with memory. Below, we start by briefly overviewing the behavioral evidence on how state curiosity enhances learning and memory. Subsequently, we lay out our arguments and speculations for each component of the PACE framework and synthesize the theoretical ideas along with the neuroscientific evidence that support our predictions.

## What Is Curiosity and How Does It Influence Learning?

One key difference between state curiosity and states associated with reward motivation is that reward delivery is generally thought to motivate future behavior that leads to the same reward. By contrast, information that completely resolves uncertainty no longer motivates exploratory behavior. Therefore, curiosity motivates us to constantly seek new, unknown information 7, 22. Consistent with this idea, initial behavioral and fMRI studies on curiosity highlight that uncertainty is a key driver of curiosity 6, 18, 23. Furthermore, the effects of reward and curiosity are not additive, and reward has been shown to undermine curiosity and its effect on memory 24, 25. Thus, curiosity can be seen as a motivational state that is at least partially distinct from states that motivate the acquisition of primary reinforcers.

Recent studies have demonstrated that curiosity significantly enhances learning and retention of information over time. Most studies that have addressed the relationship between curiosity and memory have used a trivia paradigm [15] in which participants are tested on memory for the answers to trivia questions that elicit different levels of curiosity (see Box 1 for an overview of the measurements of curiosity). These studies demonstrated that participants are better at remembering answers to questions that elicited high levels of curiosity 3, 4, 5, 15, 16, 18, 19, 25, 26, 27, 28. Curiosity-related memory enhancements are evident in immediate 5, 15, 16, 19, 26, 28 and delayed (i.e., 1 day to several weeks) 3, 4, 5, 16, 19, 25, 27 memory tests, suggesting that curiosity might enhance both memory encoding and consolidation 1, 5, 19. The effects of curiosity on memory for trivia answers does not seem to be driven by expertise or arousal, as studies suggest that curiosity-related memory enhancements are independent of the effects of prior knowledge 19, 28 and the effects of emotional arousal on memory [4]. In addition to enhancing memory in young adults, curiosity also enhances memory in healthy older adults 15, 16, 29 and in children and adolescents 30, 31.

Box 1 **Eliciting and Measuring Curiosity in the Laboratory**

**Curiosity Paradigms**

The currently dominant paradigm to elicit curiosity is a trivia paradigm in which participants encode trivia questions and their associated answers. For each question, participants usually rate their level of curiosity and confidence about knowing the answer to the trivia question. The ratings are obtained either during the encoding phase 3, 4, 15 or during a prescreening phase 5, 19. After an anticipation phase, the correct answer to the trivia question is usually shown at the end of the trial (but see [18] for a 50% probability of presentation of the answer).

In addition to the trivia paradigm that elicits epistemic curiosity, other paradigms have been recently adopted to examine perceptual curiosity and to better dissociate different processes underlying curiosity. For example, one study used blurred visual stimuli compared with clear visual stimuli to induce perceptual curiosity [23]. After an anticipation period, 50% of the stimuli were shown as clear visual stimuli. To investigate the neural mechanisms underlying perceptual curiosity, stimuli that were preceded by their associated blurry version (blurry–clear stimuli) were contrasted with images that were presented in a clear version for the whole trial (clear–clear stimuli) [23]. Another recent curiosity study used a lottery task in which participants had to anticipate different levels of monetary rewards [6]. The paradigm allows disentangling of how outcome uncertainty and information value contribute to curiosity.

**Curiosity Measures**

To measure different degrees of curiosity during a state of curiosity, most studies have relied on participants’ self-reported subjective curiosity ratings 5, 15, 16, 23, 26, 27. Other studies have operationalized curiosity in a more objective manner by measuring willingness to wait for an answer 3, 4, 6 or to sacrifice rewards (e.g., water in nonhuman primate studies, limited tokens or money in humans) 3, 20, 71 or by giving participants a choice of which stimuli they would like to experience 48, 49, 89. In general, the available evidence suggests that subjective curiosity ratings positively correlate with objective curiosity measures that implicitly test curiosity via willingness to sacrifice scarce resources 3, 4, 6. Furthermore, it has been suggested that eye-sensitive measures might be indicative of curiosity states. One study using eye tracking found that states of high curiosity, elicited by the presentation of a trivia question, were associated with participants’ anticipatory gaze toward the expected location of the answer [17] (for an oculomotor exploration task to study curiosity in nonhuman primates, see [21]). Another study showed that pupil dilations during a curiosity state are also predictive of the level of curiosity [3]. In summary, ‘willingness to sacrifice resources’, eye gaze, and pupil dilations appear to be promising objective curiosity measures in addition to self-report curiosity measures to investigate the mechanisms underlying curiosity.

Although it might seem unsurprising that people are better at learning answers to questions that piqued their curiosity, the available evidence suggests that the curiosity-related memory enhancement spreads beyond the target of a person’s curiosity. For instance, studies have used a version of the trivia paradigm in which participants were incidentally exposed to faces as they anticipated the answers to trivia questions. Although the faces were irrelevant to the trivia answers, participants showed enhanced recognition of faces learned during high-curiosity states compared with faces learned during low-curiosity states 5, 15, 19. Based on these behavioral findings of curiosity-related memory enhancements, psychological theories on curiosity, and initial neuroscientific evidence, we now lay out our arguments and speculations for each component of the PACE framework.

## Context-Based Prediction Errors: A Potential Role of the Hippocampus

Prior knowledge enables people to make predictions about the environment and, conversely, prediction errors occur in situations where there is little prior knowledge (e.g., arriving in a novel place) or when events violate one’s expectations (e.g., arriving in a familiar place to find that the furniture has been rearranged). It has been proposed that the hippocampus forms cognitive maps that allow one to generate predictions based on past experiences in similar contexts and situations [32]. Violations of hippocampally generated predictions (i.e., context-based prediction errors), in turn, were proposed to stimulate responses within a subpopulation of hippocampal neurons (termed the ‘misplace system’) that can potentially trigger exploration to resolve the uncertainty [32]. Thus, the hippocampus can be seen as playing a role in stimulating curiosity.

There is reason to think that surprising or truly novel experiences elicit curiosity. For instance, humans tend to spend more time looking at novel than familiar objects or scenes 33, 34, 35, and novelty-induced exploration appears to depend on the hippocampus 36, 37. Importantly, one study using a scene-viewing task showed that individual differences in participants’ trait curiosity predicted how well participants visually explored the novel scenes [38]. In addition, curiosity may be triggered by stimuli that are not novel *per se*, if they violate one’s expectations. For instance, people will spend time visually exploring parts of scenes that have changed relative to what they had previously seen 39, 40, and this effect also depends on the hippocampus 41, 42.

In addition to curiosity-related visual exploration, when people await the presentation of information that resolves uncertainty, eye movement behavior might also depend on curiosity. Consistent with this idea, one study using the trivia paradigm found that participants oriented their gaze earlier toward the location of an answer if the answer was anticipated with high compared with low curiosity [17]. In addition, individual variation in this earlier curiosity-triggered anticipatory gaze correlated with individual differences in trait curiosity. In summary, initial studies on curiosity using eye tracking indicate enhanced curiosity-based exploration and information seeking 7, 43, which might be elicited by context-based prediction errors dependent on hippocampal functioning [37].

## Information-Based Prediction Errors: A Potential Role of the Anterior Cingulate Cortex

Prediction errors are not limited to events in the outside world: a prediction error can also be triggered when an event violates one’s expectations about his or her knowledge on a particular topic (i.e., ‘information-based prediction errors’ or ‘information gaps’ 7, 10, 44). In an early psychological theory of curiosity, Berlyne [45] conceptualized the idea of information gaps in terms of cognitive conflict: ‘Conflicting elements or requirements often characterize the “problems” that start us off inquiring or experimenting or thinking’. Inspired by these theoretical ideas, neuroscientists proposed that the anterior cingulate cortex (ACC) is recruited during states of cognitive conflict [46]. ACC activation, in turn, stimulates recruitment of regions in the lateral prefrontal cortex (PFC) to direct actions to resolve the conflict 46, 47. Applying this concept to curiosity, we propose that the ACC might signal cognitive conflict when one is faced with an information gap and this can stimulate curiosity to find the necessary information to resolve the conflict.

Consistent with this idea, the large majority of fMRI studies on curiosity have reported activation in the ACC 5, 18, 23, 48, 49. For example, whole-brain analyses demonstrate enhanced ACC activation elicited by high- compared with low-curiosity trivia questions 5, 18 and by high compared with low perceptual uncertainty about an upcoming scene image [23]. In addition, curiosity-related ACC activity seems to be evident in curiosity paradigms in which participants act on their curiosity and choose which stimuli that they would like to see 48, 49. For example, if participants are highly curious about the revelation of a magic trick that is also associated with receiving a potential electric shock, participants show increased ACC activity when they accept the risk to satisfy their curiosity about the magic trick [48]. In addition, enhanced ACC activity seems to be evident when participants choose to see negatively valued information (i.e., morbid curiosity) compared with passively viewing the choice cue [49]. Overall, these findings support the idea that the ACC might signal information gaps due to cognitive conflicts that can trigger curiosity.

## Appraisal: A Potential Role of the Lateral PFC

In our framework, information- or environmentally triggered prediction errors might not be sufficient to trigger curiosity. Under certain circumstances these factors might have the opposite effect and induce anxiety. Relevant to this point, the hippocampus and ACC have been implicated in exploration but also in the inhibition of exploratory behavior during anxiety states 50, 51. If the hippocampus and ACC contribute to both curiosity-based exploration and anxiety-based behavioral inhibition [52], there must be an additional step that leads to curiosity and exploration.

We propose that prediction errors and information gaps trigger an appraisal process that determines one’s actions (i.e., inhibition or exploration) along with its subjective experience (i.e., anxiety or curiosity). Our framework proposes that such appraisal is supported by the lateral PFC. The importance of appraisal processes has been highlighted by behavioral studies suggesting that curiosity depends on the appraisal of one’s ability to resolve the challenges raised by the prediction error 53, 54, 55. More specifically, the appraisal is needed to determine whether the current state of uncertainty reflects a potential threat and, if not, whether one has the skills, expertise, and resources needed to resolve the uncertainty 54, 55.

In this framework, a prediction error could trigger curiosity and exploration if one feels that one has the capability to resolve the uncertainty (i.e., appraisal of higher coping potential) or it could trigger anxiety and behavioral inhibition if one believes that one has no ability to cope with the prediction error (i.e., appraisal of low coping potential). For instance, imagine that you hear a loud sound while walking in an unfamiliar neighborhood. If you think you heard the sound of a cork popping with music in the background, you might respond with curiosity and seek out the source of the sound. Alternatively, if you think the sound was a gunshot, you might respond with anxiety and rush to a safe place. In a similar vein, a student who discovers an information gap as he completes a homework assignment might become curious if he focused on the value of the necessary information on a future test. However, he could become anxious and procrastinate if he instead interpreted the information gap as a sign of his incompetence and inability to understand the topic. In these examples, we can see how an appraisal of the current situation can determine the subjective and behavioral response to a context-based prediction error or information gap.

As noted above, theories of cognitive control propose that ACC-mediated conflict signals stimulate networks in the lateral PFC that select the responses that are needed to resolve the conflict 46, 47. Substantial evidence suggests that this function can be extended to appraisal processes that mediate emotional states and emotion regulation [56]. We therefore propose that regions in the lateral PFC support appraisals that determine whether an individual will respond with curiosity in the face of uncertainty. Consistent with this idea, neuroimaging studies using the trivia paradigm have found that trivia questions associated with high compared with low curiosity showed enhanced activity in the lateral PFC potentially reflecting stronger appraisal for high- compared with low-curiosity questions 3, 5, 18, 57. Other curiosity studies also showed stronger lateral PFC activity when participants anticipated visual images with high compared with low uncertainty [23] and when participants chose to reveal an image of an unknown scene [49]. Perhaps contrary to the predictions of our framework, one study using the trivia paradigm showed that a context with highly surprising information reduced the increase of curiosity-related activation in the lateral PFC and the ACC [18]. However, further research is needed to understand how temporally extended surprising contexts relate to the sparking of item-level curiosity states.

## Curiosity Triggers Dopaminergic Neuromodulation

Substantial evidence suggests that curiosity motivates and energizes an individual to seek information to relieve that state 44, 45, 58. There are many ways that this might occur and the evidence suggests that neuromodulatory systems are likely to play an important role. Regions in the lateral PFC that we proposed to support appraisal also provide input to the dopaminergic midbrain [substantia nigra/ventral tegmental area (SN/VTA)], the origin of dopamine release 59, 60. Thus, the lateral PFC is in a position to stimulate widespread dopaminergic neuromodulation, which, as we describe below, stimulates exploratory behavior and information seeking and enhances encoding and memory consolidation 61, 62, 63.

Several models have suggested that dopaminergic neuromodulation enhances exploratory behavior along with the motivation to seek a reward 62, 64, 65. In line with these ideas and related to curiosity, ample evidence in rodents highlights the role of dopamine during exploration and, in turn, its effect on memory consolidation 66, 67, 68. For example, blockade of D1/D5 dopaminergic receptors during exploration of novel environments decreases the consolidation of new spatial maps [66], suggesting a causal link between exploration-triggered dopamine and memory consolidation. Also consistent with the idea that curiosity triggers enhanced information seeking, several studies have shown how nonhuman primates and humans have a strong preference for advance information-seeking 14, 20, 69, 70, 71. That is, if individuals have the possibility of receiving advance information about the value of an upcoming reward, they have a strong bias to select the advance information even if this option is associated with sacrificing parts of the reward. Critically, in nonhuman primates, it has been shown that such information-seeking preference for advance information is associated with enhanced dopaminergic activity in the midbrain [14].

The research on the relationship between dopaminergic activity and information seeking/exploration dovetails with prominent curiosity theories that define curiosity as a state that stimulates active exploration and information seeking to reduce uncertainty and to close information gaps 7, 44, 45, 72. Consistent with these ideas, several fMRI studies have reported that curiosity enhances activity in striatal areas that heavily depend on dopamine release by the SN/VTA 3, 5, 18, 48, 49, 57. In particular, while some studies reported activity in the dorsal striatum 3, 48, other studies showed activation throughout the striatum including the ventral striatum 5, 49. One study found that curiosity-related activity in the ventral striatum might be potentially related to the ‘tip of the tongue’ phenomenon [18], often reported as an intense feeling of ‘almost knowing’ the information and accompanied by very high curiosity [7]. Critically, studies using the trivia paradigm revealed that the dopaminergic midbrain (SN/VTA) shows increased activity for high- compared with low-curiosity trivia questions 5, 57. Another study related to curiosity revealed increased activation in the SN/VTA and ventral striatum when participants anticipated gaining information about future favorable outcomes [73]. In addition to dopaminergic activity during curiosity states, one study also found enhanced midbrain activity during the presentation of previously unknown answers [3].

## Curiosity Enhances Hippocampus-Dependent Encoding and Memory Consolidation via Dopaminergic Neuromodulation

Studies on reward and dopamine have shown that dopamine leads to an immediate attentional bias toward stimuli that have an association with past or future rewards (‘reward-based salience’) 8, 74, 75. Consistent with this, one study (also reported above) using eye tracking has shown how curiosity increases such attentional bias [17]. That is, states of high compared with low curiosity, elicited by the presentation of trivia questions, were associated with participants’ anticipatory gaze toward the expected location of the answer [17]. Further evidence for the role of increased attention in curiosity comes from neuroimaging studies that have shown that curiosity elicits activity in frontal and parietal brain areas that support attention and cognitive control 6, 23.

In line with the findings that curiosity enhances attention and that dopaminergic activity can affect immediate learning 63, 76, curiosity-related memory enhancements are evident when memory is tested immediately or after a short delay following encoding 5, 15, 16, 19, 26, 28. Neuroimaging research has demonstrated the positive influence of dopaminergic activity on curiosity-based learning. It has been shown that, when a high-curiosity trivia question is presented, curiosity-elicited activity in the ventral striatum and the hippocampus predicts curiosity-related memory enhancements for trivia answers [5]. In addition, individual differences in curiosity-based activation in the SN/VTA and hippocampus along with SN/VTA–hippocampal functional connectivity predict the magnitude of curiosity-related memory enhancements for incidental face images that are presented during curiosity states [5]. The findings are in line with the idea that curiosity enhances attentional and dopaminergic processes during learning that lead to immediate memory benefits.

In addition, prominent models and recent findings have suggested that dopamine especially enhances hippocampus-dependent consolidation 61, 63, 77, 78, 79. Consistent with these findings, curiosity-based memory enhancement for trivia answers has been shown in 12- or 24-h overnight-delayed 5, 19 and in 1–3-week delayed 3, 4, 16, 25, 27 memory tests, suggesting a role of enhanced memory consolidation for curiosity-related information. Furthermore, dopamine-related ‘tag-and-capture’ models suggest that novelty-mediated increases in dopamine availability enhance the retention of memories for events that occur before or after the novel event takes place 61, 62, 80. These ideas and findings align well with recent findings in curiosity research that show enhanced retention of incidental material that was presented during high-curiosity states 5, 19. Consistent with the role of enhanced hippocampal consolidation via dopaminergic modulation, it has recently been shown that intrinsically regulated learning (potentially related to curiosity) engages SN/VTA–hippocampal processes and in turn promotes consolidation-related enhancements 81, 82.

## How a PACE Cycle Influences Memory

Having laid out the proposed components of the PACE framework along with the supporting evidence, we are in a position to better understand how curiosity shapes learning and memory. As we have outlined, it becomes apparent that there is not a single factor that elicits curiosity and no single process through which curiosity affects memory. Instead, there are several factors that trigger curiosity and curiosity affects memory in multiple ways. In general, our framework provides a model of how external, context-based and internal, information-based prediction errors – supported by the hippocampus and ACC, respectively – stimulate appraisal in the lateral PFC. Such lateral PFC-related appraisal then leads to either anxiety/inhibition related to amygdalar processes [52] or curiosity/exploration related to dopaminergic processes [1]. If curiosity is sparked, curiosity enhances learning via increased attentional processes 3, 5, 17 and retention via enhanced memory consolidation 4, 5, 19. The aspect of the PACE framework of how curiosity shapes learning and memory via dopaminergic functions is in line with theoretical models on how dopaminergic activity enhances hippocampus-dependent encoding and memory consolidation 61, 62, 63. Importantly, the PACE framework extends these previous models and proposes how lateral PFC-related appraisal mediates how hippocampus- and ACC-related prediction errors trigger curiosity-related neuromodulation. Although our proposed framework focuses on state curiosity and its effect on memory, the individual components of the PACE framework might also be influenced by individual variation in trait curiosity (Box 2).

Box 2 **Trait Curiosity and the PACE Framework**

Although our framework focuses on state curiosity and its effects on memory, prior research has focused primarily on trait curiosity – the tendency to experience curiosity as a stable personality characteristic (for a review see 1, 2). Importantly, theories of curiosity have suggested a link between trait and state curiosity. That is, individuals who show higher trait curiosity engage in enhanced information seeking and curiosity-based exploration 7, 43. In line with this idea, recent experimental studies have also emphasized how individual differences in trait curiosity affect curiosity-driven exploration 70, 90. Within our framework, it is possible that the degree of involvement of the proposed PACE components might rely on individual variation in trait curiosity (Box 3).

For the context- and information-based prediction error components, it might be possible that individual differences in trait curiosity differently elicit hippocampus- and ACC-dependent prediction errors. Potentially consistent with this idea, the degree of curiosity-based exploration measured via eye movements 17, 38 – potentially underlying hippocampus-dependent context-based prediction errors [37] – has been shown to correlate with trait curiosity. In addition, the degree of information-seeking behavior in an experiment in which participants searched different Wikipedia articles also correlated with trait curiosity; specifically, the particular aspect of trait curiosity that is related to closing an information gap (i.e., deprivation sensitivity) [90]. Therefore, relationships between trait curiosity and curiosity-based behavior might rely on individual differences in the sensitivity to hippocampus- and ACC-related prediction errors.

Regarding the appraisal component, appraisal of the current situation will depend not just on the current state of uncertainty but also on a person’s trait curiosity. It has been shown that individual differences in the strength of appraisal (i.e., how well a person can cope with the uncertainty) explains the individual differences in trait curiosity [53]. That is, ‘curious people appear to be curious because they are more likely to appraise their ability to understand as high’ ([53], see p. 108). Consistent with this finding, a series of experiments has shown that participants with high coping potential show higher curiosity/interest in complex novel products and inventions [55], suggesting that appraisal leads to curiosity only if the prediction errors seem manageable.

Does the curiosity component, which triggers information seeking and exploration, potentially depend on individual differences in trait curiosity as well? Here, it might be relevant to consider that individual differences in the more general personality trait ‘openness to experience’ – which encompasses curiosity as a critical subcomponent – has been linked to dopaminergic functions 91, 92. This suggests that dopaminergic functions also extend to ‘cognitive exploration’. Consistent with this relationship between dopamine and cognitive exploration, it has been shown that the strength of a white matter connection between dopaminergic regions and the hippocampus 93, 94 (i.e., via the fornix) correlates with the degree of trait curiosity [95].

Regarding the final PACE component of how curiosity enhances memory, it has also been shown how trait curiosity enhances real-world learning 96, 97, 98. For example, it has been shown that trait curiosity mediates the effect on learning during a medical training program [97]. In addition, trait curiosity predicts academic performance even if the effects of intelligence and effort on academic performance are controlled [96].

Within our framework, a PACE cycle will be completed once uncertainty is resolved and curiosity is satisfied by closing an information gap. However, in many cases the presentation of the information that was associated with curiosity might elicit a further context- or information-based prediction error. In line with this idea, several studies using the trivia paradigm demonstrate how prediction errors triggered by the trivia answers themselves benefit memory 4, 16, 18, 27, 30. Within our framework, we propose that such further prediction errors will start a new PACE cycle, which could then further benefit memory and promote knowledge acquisition [83].

Critically, new paradigms will need to be developed to test several predictions of the PACE framework. For example, future neuroimaging studies would need to investigate how active exploration and information seeking (rather than the passive awaiting of information associated with different levels of curiosity) enhances curiosity-related memory. Furthermore, it will be central to rigorously test the proposed role of each component within the PACE framework and how the proposed components functionally interact with each other in support of curiosity-based memory. In Box 3 we outline the currently most relevant predictions of the PACE framework.

Box 3 **Currently Most Relevant Predictions of the PACE Framework**

**Prediction Error Components Supported by Hippocampus and ACC**

(i) Larger context- and information-based prediction errors elicit increased activity in the hippocampus and ACC, respectively.
  (a) Recruitment of specific hippocampal regions depends on the functions along the longitudinal axis of the hippocampus (from gist-based to detailed information in the anterior to posterior hippocampus, respectively).
(ii) The two types of prediction error are elicited in parallel but depending on the situation one type of prediction error may play a larger role than the other.
  (a) Unexpected changes in the environment (e.g., surprise in the environment) relies more on hippocampus-dependent prediction errors.
  (b) Information that elicits higher cognitive conflict (e.g., ambivalent information, incongruent information to prior knowledge) relies more on ACC-dependent prediction errors.
(iii) To support successful appraisal, larger prediction errors result in stronger hippocampal–lateral PFC and ACC–lateral PFC coupling for context- and information-based prediction errors, respectively.

**Appraisal Component Supported by Lateral PFC**
(i) Enhanced recruitment of lateral PFC in situations that require more appraisal (e.g., situations that introduce a choice or even a dilemma about whether to explore or to abstain from information).
(ii) The lateral PFC will appraise information related to hippocampus- and ACC-dependent prediction errors and mediate the effect of prediction errors to elicit curiosity or anxiety.
(iii) Individual differences in coping strategies affect lateral PFC-related appraisal and the subsequent elicitation of anxiety or curiosity. For example, appraisal in individuals with higher stress tolerance might be more likely to elicit curiosity than anxiety.
(iv) Subpopulations and patients with suboptimal PFC functions rely less on appraisal in eliciting curiosity. Such suboptimal appraisal mechanisms may lead to altered curiosity (e.g., more inconsistent curiosity). For example, less developed PFC functions in children may lead to differences in curiosity (less mediated by appraisal and potentially more variable) compared with adults.

**Curiosity Component Supported by SN/VTA and Striatum**
(i) Exploratory behavior and eye movements related to curiosity are supported by activity in the ventral striatum and SN/VTA.
(ii) The strength of lateral PFC–SN/VTA functional connectivity predicts the degree of curiosity and exploratory behavior. By contrast, lateral PFC–amygdala functional connectivity predicts whether appraisal leads to anxiety-related behavioral inhibition.
(iii) Dopaminergic functions within the striatum and SN/VTA vary across individuals in the healthy population and are decreased in older adults and in certain clinical conditions (e.g., Parkinson’s disease, schizophrenia, depression, Alzheimer’s disease). Such alterations in dopaminergic functions lead to decreased levels of curiosity and exploratory behavior.

**Enhanced Encoding and Consolidation Component Supported by Hippocampus**
(i) Curiosity-related eye movements and exploratory behavior predict curiosity-based memory enhancements.
(ii) While memory enhancements for curiosity target information (e.g., trivia answers) might depend on hippocampal processes both during encoding and during consolidation, memory enhancements for incidental information (i.e., unrelated information that is temporally contiguous to a curious state) depend more on enhanced consolidation than encoding processes.
(iii) Individual variability in functional and structural connections between the hippocampus and dopaminergic regions predicts curiosity-based memory enhancements.
(iv) Alterations in dopaminergic functions in subpopulations and certain clinical conditions (e.g., Parkinson’s disease, schizophrenia, depression, Alzheimer’s disease) decrease the positive effects of curiosity on hippocampus-dependent memory.

## Concluding Remarks

In recent years there have been exciting developments in the study of curiosity and its impact on memory. Drawing from converging evidence in psychology and neuroscience, we proposed the PACE framework, which integrates theories about curiosity with existing models on prediction errors, appraisal, exploration, and the neuromodulation of hippocampus-dependent memory.

At this point, it is important to point out that we do not mean to imply that the above framework captures all of the relevant processes and experiences. Moreover, we are certain that the underlying neural substrates of curiosity-related memory enhancements are much more complex than what we have proposed. At the same time, the PACE framework provides a starting point for understanding how curiosity can influence memory by bringing together a wide range of findings and by specifying potential links across multiple levels of analysis (i.e., process, neural, behavioral, and subjective level). We believe that the PACE framework will be useful for the emerging research field of curiosity and memory to stimulate future research (Box 3; see Outstanding Questions).

Understanding the neural mechanisms of how curiosity enhances learning is likely to be of interest in the rapidly expanding field of artificial intelligence 84, 85 as it might be a critical approach to stimulate independent, curiosity-guided learning in artificial systems. Furthermore, future research on curiosity and memory has potentially far-reaching implications for education 86, 87, 88; that is, to guide policymaking and to inform teachers of how curiosity can be harnessed in the most effective way in the classroom. It would therefore be promising to test how laboratory-based findings on curiosity and memory translate to applied settings (see Outstanding Questions).

Outstanding Questions

What are the relationships between curious states and curiosity traits and how do they jointly or independently influence learning and memory?
How does curiosity interact with post-information processes in support of learning (e.g., surprise, interestingness, novelty)?
How does curiosity interact with emotional effects on memory?
How does curiosity affect early and late memory consolidation during awake rest and sleep periods?
What are the boundary conditions of curiosity-related memory enhancement for incidental information that is temporally contiguous to a state of high curiosity?
How does curiosity affect learning and memory across the lifespan?
How does curiosity influence memory in patients with memory impairments?
Do curiosity-related memory enhancements depend solely on dopamine or also on other neurotransmitters (e.g., noradrenaline or acetylcholine, which have been proposed to support different types of uncertainty)?
Can we translate laboratory-based findings on curiosity-related memory enhancements to the classroom and clinical settings?
How can the findings on the neural mechanisms of curiosity-based learning be translated for artificial intelligence/robotics applications?
